# Grape Waste Materials—An Attractive Source for Developing Nanomaterials with Versatile Applications

**DOI:** 10.3390/nano13050836

**Published:** 2023-02-23

**Authors:** Anda Maria Baroi, Elwira Sieniawska, Łukasz Świątek, Irina Fierascu

**Affiliations:** 1 National Institute for Research & Development in Chemistry and Petrochemistry—ICECHIM, 060021 Bucharest, Romania; 2 Faculty of Horticulture, University of Agronomic Sciences and Veterinary Medicine of Bucharest, 011464 Bucharest, Romania; 3 Department of Natural Products Chemistry, Medical University of Lublin, 1 Chodzki, 20-093 Lublin, Poland; 4 Department of Virology with SARS Laboratory, Medical University of Lublin, 1 Chodzki, 20-093 Lublin, Poland

**Keywords:** grape waste, phytochemicals, nanotechnology, metal nanoparticle, healthcare applications

## Abstract

In the last decade, researchers have focused on the recycling of agro-food wastes for the production of value-added products. This eco-friendly trend is also observed in nanotechnology, where recycled raw materials may be processed into valuable nanomaterials with practical applications. Regarding environmental safety, replacing hazardous chemical substances with natural products obtained from plant wastes is an excellent opportunity for the “green synthesis” of nanomaterials. This paper aims to critically discuss plant waste, with particular emphasis on grape waste, methods of recovery of active compounds, and nanomaterials obtained from by-products, along with their versatile applications, including healthcare uses. Moreover, the challenges that may appear in this field, as well as future perspectives, are also included.

## 1. Introduction

Due to population growth, rapid modernization, and globalization, the amount of food waste from industries and households is increasing. Agro-industrial waste is usually discarded in landfills, contributing to environmental issues. The volume of food waste generated globally is higher than 1.3–1.4 billion tons and is expected to increase up to 2.6 billion tons by 2025 [1], with an economic loss of about USD 400 billion [2]. Hence, it is necessary to implement different approaches to transform agro-industrial and food waste into value-added products [3].

Recently, the interest in obtaining natural ingredients from food waste has increased significantly. These components could be transformed into value-added products, such as food ingredients, nutraceutical compounds, or biodiesel, utilized by a broad range of industries [4]. Importantly, agricultural waste, instead of ending up in landfills and generating environmental hazards, can be cost-effectively turned into sustainable resources [5,6,7]. The valorization of plant- and animal-origin by-products provides opportunities to obtain value-added products, thus minimizing the environmental impact and promoting and commercializing new materials rich in bioactive compounds [8,9,10]. An example of such an approach is the utilization of wastes generated by grape production and the wine industry.

The practice of viticulture plays a significant role in the effective development of production sectors aiming to obtain grapes specific for each industry. Grapevine pruning, necessary for balancing growth and development, is the primary source of viticulture waste. Since viticulture is one of the most common agricultural practices worldwide, it leads to a significant accumulation of biomass waste, such as shoots, canes, and leaves, amounting to between 6–18 million tons annually [11]. Over time, wine has become one of the most consumed drinks globally, according to the International Organization of Vine and Wine [12], reaching a consumption rate of almost 230 million hectoliters in 2021. However, massive amounts of grape pomace are generated as a result of the winemaking process. Grape pomace is made up of different ratios of seeds, skins, stalks, and wine lees, depending on the grape variety, fermentation, and pressing processes [13]. The valuable resources available in grapevine waste biomass and grape by-products, such as cellulose, hemicellulose, lignin, minerals, and bioactive compounds [14], can be used in different industries, contributing to the development of an efficient circular economy.

Nanobiotechnology is based on applying nano-techniques in the fields of biological and medical sciences to improve human health and quality of life. From its beginning in 1959, the development of nanotechnology as a multidisciplinary field of science has led to the discovery of a significant number of nanomaterials with practical applications in the pharmaceutical industry and medical sciences. These include innovative drugs and drug-delivery systems, biosensors, bioimaging gene-delivery techniques, and innovative nutraceuticals [15].

The recovery of bioactive compounds and their valorization in different industries creates an excellent opportunity to reduce environmental impact, promote the circular economy, reduce food waste, and obtain novel ingredients with potential health benefits [16]. Therefore, this paper aims to critically discuss plant waste, with particular emphasis put on grape waste, methods of recovery of active compounds, nanomaterials obtained from by-products, and their versatile applications, including healthcare uses. Moreover, the challenges that may appear in this field, as well as future perspectives, are also included.

## 2. Recovery of Active Compounds from Grape Waste and Their Beneficial Effects

Agriculture-originating wastes contain a mixture of natural products, including phenolic compounds, carbohydrates, polysaccharides, and lignocellulose. Recovery of these compounds involves specific treatments, but results in a significant reduction of economic costs affecting other industries, along with reduced environmental impact [17].

Agro-wastes result from the processing crop or animal products for human use and include the by-products and leftovers from the food processing industry [18]. Pomace, peels, molasses, or shells can be considered recyclable wastes, which, after the application of appropriate pretreatment methods, can be processed into various products or raw materials for use in other industries. These methods are often material-specific, each presenting advantages and disadvantages (Table 1). As a modern pretreatment method, ohmic heating (OH) was studied by Pereira et al. [19] for the aqueous extraction of total soluble matter and phenolic compounds from red grapes. Given the thermal nature of OH to generate internal heat, it was possible to establish a direct flash-heating extraction without promoting the thermal degradation of the molecules of interest, obtaining a recovery yield two times greater than that of the control sample (1348.8 µg/g compared with 756.2 µg/g) [19].

**Figure 1:**
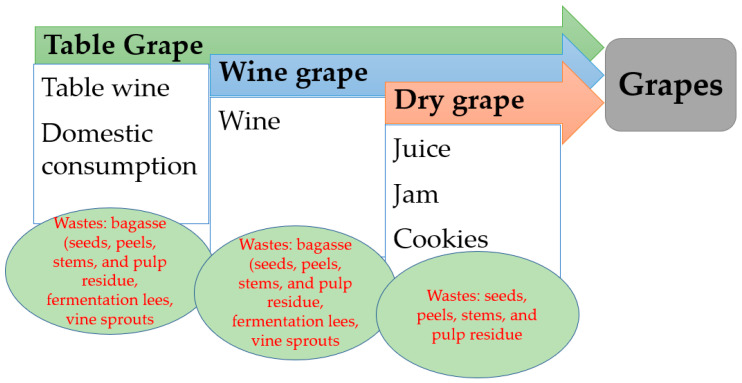
Forms of grape consumption and the obtained residues.

Grapes are one of the most widely grown fruits in the world. There are three distinct purposes of grape production: grapes for the wine industry, table grapes, and dried grapes (raisins) (Figure 1) [25].

The morphology of grapevine consists of vegetative and reproductive organs. The first category includes leaves, tendrils, shoots, trunks, and roots. The second is represented by clusters with flowers or grapes [26].

Different levels of bioactive compounds are found in various plant parts of the grapevine that are commonly considered and treated as viticultural waste. Scientific studies of grapevine leaves indicated a relatively low content of hydroxycinnamic acids, stilbenes, and hydroxybenzoic acids. Conversely, a high concentration of flavonols, representing the most abundant phenolic compounds, was found [27]. Thus, phytochemicals often found in grapevine leaves, presented in decreasing quantitative order, are as follows: quercetin-3-O-glucuronide > quercetin-3-O-galactoside > quercetin- 3-O-glucoside > caftaric acid > quercetin-3-O-rhamnoside > kaempferol-3-O-glucoside > kaempferol-3-O-glucuronide > coutaric acid > myricetin-3-O-glucoside > quercetin-3-O-rutinoside > and kaempferol-3-O-rutinoside [28]. In the case of shoots, bioactive compounds found in concentrations of over 500 mg/kg, listed in decreasing quantity, are: trans-resveratrol > (+)-trans-ε-viniferin > catechin > sinapic acid > isohopeaphenol > vitisin B > trans-piceatannol > trans-ω-viniferin > hopeaphenol > procyanidin B1 [29]. Furthermore, the stilbene compounds are found in the roots and trunks of the grapevine, namely: (+)-trans-ε-viniferin, (+)-cis-ε-viniferin, isohopeaphenol, α-viniferin, and trans-resveratrol [30].

Grape pomace, the solid residue formed after the pressing and fermentation of grapes, is the most representative waste from winemaking [31]. This winey residue is mainly comprised of grape seeds, skins, pulp, and stalks [32]. Its quantity depends on the variety of grapes, the nature of the soil, and the processes and equipment used in the winemaking process [33]. The Italian Central Statistics Institute reported that more than 80% of the total production of grapes is intended for the winemaking process. It corresponds to more than 7 million tons of grapes per year, yielding about 1 million tons of pomace [34]. It was shown that the total polyphenolic content in the grape pomace is between 5 and 6 g of gallic acid equivalents (GAE)/100 g [35]. Proteins constitute 5–14 g/100 g of pomace, and lipids make up 1–13 g/100 g [36], whereas cellulose, hemicellulose, and lignin comprise 7–9 g/100 g, 6–22 g/100 g and 11–23 g/100 g, respectively [37]. The bioactive compounds often found in grape pomace, but varying in different grape fractions, are phenolic acids, stilbenes, flavonols, anthocyanins, and proanthocyanidins. Proanthocyanidins and flavan-3-ols, especially (+)-catechin, (−)-epicatechin, procyanidin B1, and procyanidin B2, are the primary compounds in grape seeds [38,39]. At the same time, grape skins show high levels of anthocyanins [40], flavonols, such as quercetin, myricetin, and kaempferol [41], along with the most important grape stilbene, namely resveratrol [42].

The beneficial human health properties of the compounds mentioned above are well known. The anti-inflammatory effect of Negrano grape pomace polyphenols was demonstrated on intestinal cells by controlling the crucial mediators of the oxidative and inflammatory process, namely pro-inflammatory cytokines, chemokines, adhesion molecules, and matrix metalloproteinases. Calabriso et al. [43] extracted the powdered grape pomace with methanol/ethanol (80:20, *v*/*v*) and subjected the extract to phytochemical and biological analysis. Different levels of gallic acid, catechin, and quercetin were found, but the most representative bioactive compounds were oenin and epicatechin, amounting to 4.9 and 3.7 mg/g, respectively. The extract dose-dependently inhibited the release of interleukin (IL)-6, monocyte chemoattractant protein (MCP)-1, and matrix metalloproteinases (MMP)-9 and MMP-2 from enterocyte-like cells. Additionally, gene expression of pro-inflammatory markers, namely IL-1β, TNF-α, macrophage colony-stimulating factor (M-CSF), C-X-C motif ligand (CXCL)-10, intercellular adhesion molecule (ICAM)-1, vascular cell adhesion molecule (VCAM)-1, and cyclooxygenase (COX)-2 was decreased [43]. In a study conducted by Leal et al. [44], polyphenolic compounds belonging to hydroxybenzoic and hydroxycinnamic acids, flavanols, stilbenes, and anthocyanins were identified and quantified. Catechin was the primary compound in grape stem extracts, with a 0.44–2.03 mg/g dry weight. The extracts showed significant antioxidant activity and antimicrobial properties against *S. aureus* and *E. faecalis*. They also exerted anti-inflammatory activity by inhibiting the production of NO at non-toxic cellular concentrations and by inhibiting tyrosinase and elastase activity, thus suggesting the possible application of these extracts in cosmetic products [44]. Furthermore, Sangiovanni et al. [45] indicated that grapevine leaf extracts could inhibit and alleviate gastric inflammation by affecting the NF-κB pathway. The aqueous extract of vine leaves was characterized by the HPLC-DAD method, showing the presence of flavonols, caffeic acid derivatives, and anthocyanins. The contents of total phenolics of 146.3 mg GAE/g and 4.3 mg/g of anthocyanins (expressed as cyanidin-3-glucoside equivalents) were reported. The inhibitory effect of the extracts on IL-8 secretion was examined in gastric and intestinal epithelial cells after gastrointestinal digestion [45]. Thus, the grape pomace polyphenols may exert multiple health-promoting properties, potentially decreasing or preventing gut chronic inflammatory diseases and improving vascular endothelial function [43,45].

**Figure 2:**
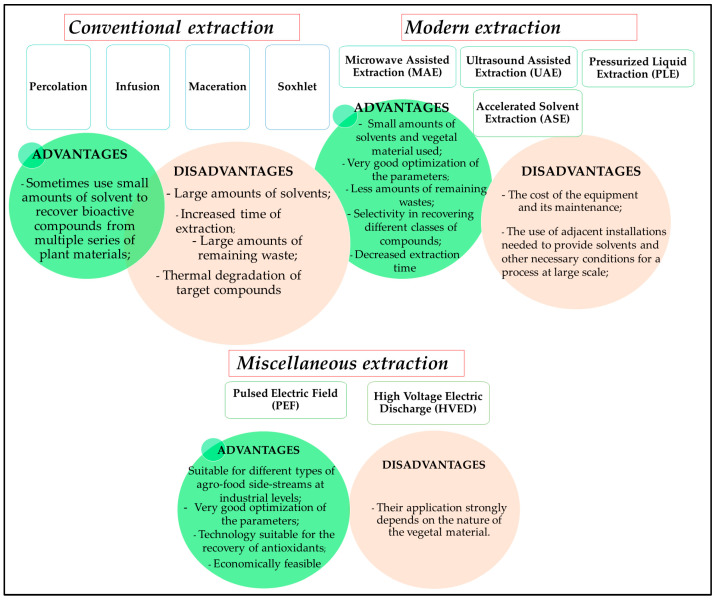
Schematic presentation of the extraction methods.

Numerous extraction methods were developed to recover valuable bioactive compounds from viticulture and winery wastes for application in different industries and circular economies [46]. The traditional methods, including Soxhlet extraction, maceration, or reflux extraction, require large amounts of solvent and energy, making these methods unsuitable for obtaining extracts for commercial uses [47,48]. However, modern techniques, such as supercritical carbon extraction, ultrasound-assisted extraction, accelerated solvent extraction, pressurized fluid extraction, and microwave-assisted extraction, have gained new valences in recent years. They provide numerous advantages, including short extraction time, small amounts of solvents, increased yields of extraction, and cost-effectiveness [49]. Moreover, methods based on the electric field require less processing time and energy consumption and are suitable for reducing the degradation of heat-sensitive compounds [50]. A schematic representation of the advantages and disadvantages of these methods is presented in Figure 2.

The ultrasound assisted extraction of grape pomace polyphenols was performed at 100 °C for tannins, and at 150 °C for tannin-anthocyanin fraction. Interestingly, a decrease in polyphenol yield was observed at longer extraction times and higher temperatures, before and after the enological fermentation of pomace [51]. The pulsed electric field extraction is one of the most efficient techniques used to enhance the yield of target compounds and improve the quality of juices [52]. For the hydroethanolic extracts of grape seeds, Boussetta et al. observed an increase in the yield of total phenolic content from 16.7% to 84.2% after the application of electrical treatments [53]. Combining pulsed electric field extraction with ultrasound technology, Ntourtoglou et al. promoted the extraction of volatile compounds and polyphenols from grape stems [54].

Among other miscellaneous extraction techniques, pulsed ohmic heating and high-pressure processing are also known to be suitable for the extraction of thermolabile compounds [13]. The yield of anthocyanins from red grape pomace ethanolic extract obtained with the assistance of the high hydrostatic pressure was 50% at 600 MPa and 70 °C [55]. The antioxidant effect was not directly correlated with the highest amount of anthocyanins, which were optimally extracted at 100% ethanol, 50 °C, and 600 MPa. However, the extraction yields were about 23% higher than those under control conditions. For extracting target compounds from grape wastes, the primary objective of the technologies used was to gain a faster extraction rate with an increased extraction and recovery yield, with lower energy and solvent consumption. It was observed through the analysis of antioxidant activity and the total phenolic content that the extraction kinetics were enhanced by increasing the temperature and maintaining the optimal solvent condition [56].

Besides phenolic compounds, dietary fibers represent another class of interest for recovery from grapes wastes. Their beneficial effects on human health, such as reducing the risk of cardiovascular diseases; protection against cancer, diabetes, and blood cholesterol; or the prevention of obesity, were described [57]. The main products resulting from grape processing are rich sources of dietary fibers: grape skins are lignocellulosic materials containing hemicellulosic sugars that, after hydrolysis, produce solutions containing a wide variety of xylose and glucose monomers [58], whereas grape pomace is a source of monosaccharides [59]. Different studies reported the content of dietary fibers from 50 to more than 80% of dry matter, with no significant differences between red and white grapes [60,61]. Thus, the interest in recovering such compounds is increasing, and the development and optimization of recovery technologies is an excellent alternative for obtaining value-added products. The most commonly used method is conventional hot water extraction, with low energy resource and solvent requirements, and which can be upgraded using modern techniques, such as ultrasound technologies, or by applying enzymes to help disrupt the cell wall structure [62]. Another highly efficient and environmentally friendly pretreatment technology is the steam explosion method, which can be used as a pretreatment method to obtain bioactive compounds. Cui et al. demonstrated that steam explosion could convert insoluble dietary fiber from grape pomace into soluble dietary fiber, while increasing oil-holding and sodium nitrite binding capacity at optimal conditions of 0.8 MPa for 3 min [63]. Micronization can affect dietary fiber’s solubility and hydration properties, enhancing the release of phenolics [64]. Moreover, enzymatic treatment can disrupt the grape pomace cell wall by breaking glycosidic linkages from polysaccharide chains into mono- and oligosaccharides [65].

Lipids, found in grape seeds, are also of great interest. The recovery of fatty acids, tocopherols, tocotrienols, and phytosterols from grape seed oil is one of the most popular applications [66]. Linoleic, oleic, and palmitic acids were found in Bulgarian grape varieties [67], whereas myristic, palmitoleic, margaric, arachidic, nanodecylic, paullinic, behenic, and lignoceric acids were obtained from grape cultivars in China [68]. Their recovery involves obtaining grape seed oil, generally using traditional cold screw pressing [69], solid-liquid extraction using different solvents, supercritical fluid extraction, ultrasound extraction, [70] or enzymatic hydrolysis [71].

## 3. Potential Applications of Grape Wastes

### 3.1. Phenolic Compounds Obtained from Grape Wastes Used for Nanotechnological Formulations

**Figure 3:**
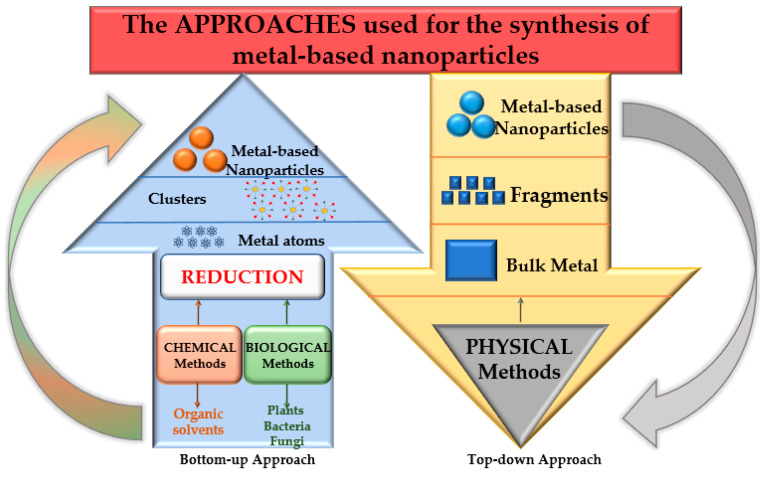
Bottom-up and top-down approaches for NP synthesis.

Plant-derived materials are expected to become effective therapeutic modalities for disease treatment, drug delivery, or other applications (environmental, food packaging, etc.) that can increase the quality of life. Nanomaterials such as metallic nanoparticles can be synthesized by bottom-up and top-down approaches from molecules or atoms to bulk precursors (Figure 3).

**Figure 4:**
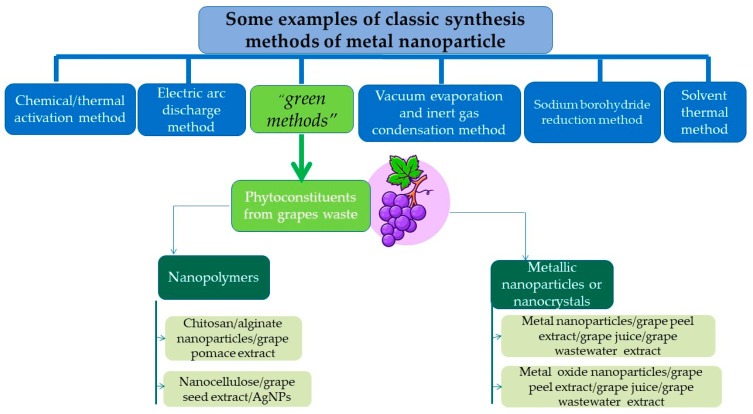
Methods of preparation of different nanomaterials, including examples of grape waste use. (Designed by Brgfx/Freepik).

The top-down approach covers the destructive methods used to reduce the bulk material to nanoparticles (mechanical milling, nanolithography, laser ablation, sputtering, and thermal decomposition), whereas the bottom-up approach, or the constructive techniques, are used to build up the material from atoms (sol-gel method, spinning, chemical vapor deposition, pyrolysis, ionizing radiation assisted synthesis, biosynthesis) [72]. The synthesis methods gathered under the term “*biosynthesis,*” or “*green methods,*” are of particular interest for obtaining nontoxic nanoparticles [73]. Under the large biosynthesis class, the most encountered methods use bacteria, fungi, or plant extracts, along with the precursors, instead of conventional chemicals for bio reduction and capping purposes. Metal nanoparticles obtained by biosynthesis using plants represent noble metal nanoparticles or common metallic nanoparticles. Different types of plant extracts can be used in order to obtain various compositions, sizes, shapes, and controlled polydispersity [74]. These methods reduce the use of hazardous chemical reagents and have been proven to be safe and economically sustainable alternatives. Despite the advantages, a critical step in synthesising metal nanoparticles mediated by plant extracts is their stabilization, mainly to prevent agglomeration and oxidation processes. Phytoconstituents recovered from grape waste extracts can act as reducing and capping agents, allowing for the control of the size and morphology of the obtained nanoparticles [75]. Figure 4 presents the preparation methods of different nanomaterials, including the exemplary application of grape waste.

#### 3.1.1. Metallic Nanoparticles

**Figure 5:**
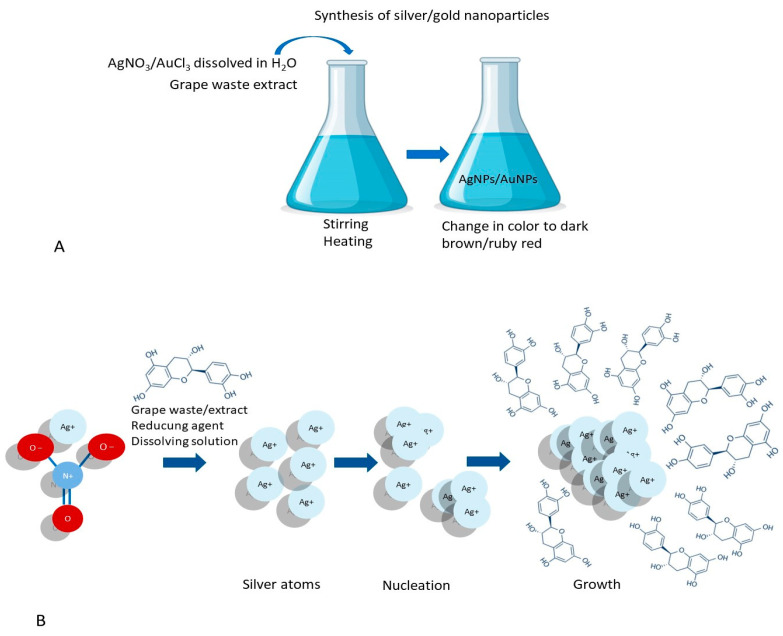
The synthesis of metal nanoparticles based on grape waste: (**A**) a general procedure; (**B**) the physicochemical process of forming metal nanoparticles. (Designed by Brgfx/Freepik).

The use of natural compounds in biological synthesis is an excellent alternative to classical (physical or chemical) methods used in nanomaterial production [76]. Fruit wastes represent a significant source of municipal waste. These waste have been evaluated as reducing and capping agents in the synthesis of nanoparticles (NPs). Due to the abundance of phenolic compounds, fruit waste extracts possess the property of high nucleophilicity and can interact with metallic ions through chelation [77]. Moreover, they can reduce metallic salts to metallic nanoparticles [78]. Figure 5 presents the synthesis of metal nanoparticles based on phytoconstituents from grape waste.

**Figure 6:**
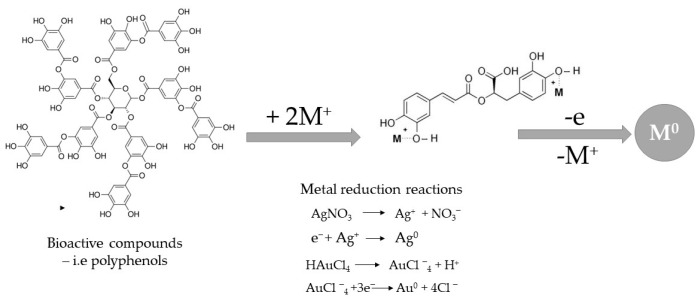
Proposed mechanism for obtaining metal nanoparticles via plant extracts.

The exact mechanism of the reduction of metallic ions to metallic nanoparticles using plant extracts is still under debate. The proposed approaches are based on the use of different active compounds, including polyphenols, flavonoids, and other bio-active compounds found in plant material (Figure 6).

Due to the complex composition of plant extracts, it is impossible to assign a particular active compound as the primary reducing agent; it is probable that a combination of various biomolecules found in natural extracts plays a reducing and stabilizing role in a synergic manner [79]. Obtaining these nanomaterials meets the demand for resource recycling with practical benefits, no matter what source is used (medicinal and aromatic plants, agro-food waste, or non-eatable plants), depending on the availability of material acquisition and considering the simplicity of the extraction process [78,79]. The concentration of the recovered compounds, which has a powerful influence on nanomaterial formation, can be a factor.

The *green materials* (obtained without hazardous chemicals) can be used for different applications: medical, environmental, or industrial. Phytoconstituents (such as phenolic compounds) can form metal-based nanoparticles, alloy nanoparticles, or metal oxide nanoparticles. However, different parameters, such as pH, contact time, solvent, reaction temperature, and method adopted, which can tailor the preparation and final application of nanomaterials, must be considered.

Natural compounds obtained from waste can be used to sustainably maintain the postharvest quality of the same type of plant that generated the waste. This was presented in the case of grape seed extract (GSE). To maintain the quality of fresh grapes, Zhao et al. [80] developed a formulation using grape seed extract-silver nanoparticles for food packaging. The developed composite of chitosan/AgNP-grape seed extract significantly reduced decay percentage, total yeast and mold counts, and weight loss, while maintaining the titratable acidity of grapes. GSE was used as a capping and reducing agent in the synthesis of AgNP. The material was used successfully against *Escherichia coli* O157:H7 and *Listeria monocytogenes* [80]. For the same purpose, Soto et al. [81] used grape peel leftovers from winemaking. They proposed the green synthesis of silver nanoparticles with antimicrobial activity against foodborne pathogens [81]. Montagner et al. [82] developed a hydroalcoholic extraction method to obtain high concentrations of bioactive compounds from ground grape seeds of the Merlot variety originating from wine production residues. Following the phytochemical tests, the authors reported a total phenolic content ranging from 418.30 to 1473.86 μg of GAE/mL of extract, 387.08–1000.63 μg of catechin equivalent per ml of extract for flavonoids, and 0.14–0.59% for catechins. These grape seed extracts were also proposed for reuse in developing nanotechnological products with high added value, such as new functional food ingredients and sustainable packaging [82]. In another study, gold nanoparticles (AuNPs) were phytosynthesized; the reducing agent, in this case was the aqueous pomace extract. The results demonstrated that AuNPs exhibit antioxidant and tyrosinase inhibitory properties, as was evaluated using ABTS and enzymatic assay. In addition, in vitro assays demonstrated the ability of these NPs to alleviate H_2_O_2_-induced growth inhibition, exhibiting scavenging activity against intracellular reactive oxygen species. The ability of AuNPs to screen solar radiation due to the organic layer present on their surface was confirmed for up to 200 min. All these data suggested that AuNPs, obtained with pomace extracts, can be proposed in nanomedicine and cosmetics as an anti-aging ingredient for skin care products [83]. Silver nanoparticles developed by Saratele et al. presented significant free radical scavenging activity against DPPH (IC_50_, 50.0 ± 2.25 µg/mL) and ABTS radicals (IC_50_, 38.46 ± 1.14 µg/mL), as well as the inhibition of carbohydrate hydrolyzing enzymes, along with antibacterial properties [84]. Moreover, grape pomace tannins were found to form silver nanoparticles, showing antidiabetic and antioxidant potential, as well as antimicrobial activity [85]. Table 2 presents the application of grape waste in NP formulations.

#### 3.1.2. Polymeric Nanocomposites

Nanocomposites are of particular interest because of their special composition based on nano-sized reinforcing agents. These agents were proven to be effective in enhancing the barrier and mechanical properties of biopolymers due to their high matrix/filler interfacial area, with the internal layer of the composite materials usually acting as a “transport vector,” releasing the active compounds [91]. These composite materials possess valuable properties, such as lower toxicity risks, biocompatibility, biodegradability, recyclability, and tunable surface features, for use in healthcare applications. Wu et al. [89] reported that nanocellulose/grape seed extract/AgNPs composite film has a lower antibacterial effect against *E. coli* than that observed against *S. aureus* (diameter of inhibition zone (mm)—approx. 2 mm and 6 mm, respectively). These results were probably attributable to the structural differences in the cell wall of bacteria, with *E. coli* (Gram-negative bacteria) having a multilayer structure, based on a peptidoglycan layer, a lipoprotein layer, and a phospholipid or lipopolysaccharide layer, thus not allowing for the optimal penetration of AgNPs [89]. Grape pomace extract (GPE) produced by enzymatic extraction followed by freeze-drying, encapsulated into chitosan and alginate nanoparticles, was demonstrated by Costa et al. [90] to be a good candidate for oral delivery applications. They presented permeability studies across a Caco-2/HT29-MTX co-culture model, demonstrating that the encapsulation of GPE decreased the permeability of xylobiose, thus offering a beneficial retention time in the intestine and potentially improving the prebiotic potential of grape pomace extract [90].

In addition, these materials can be used as main ingredients in the food packaging industry. Biopolymers, such as starch, represent a challenge for food packaging because bacteria may metabolize them. With an enhanced demand for biodegradable packaging, there is a need to functionalize the biopolymers with bioactive compounds to prevent bacterial and fungal attacks [92]. Xu et al. incorporated grape pomace extract into tapioca starch active nanocomposite films for ready-to-eat chicken meats, proving an antibacterial activity against *L. monocytogenes* inoculated onto the meat samples during the 10-day storage period at 4 °C [93].

#### 3.1.3. Lipidic Materials

Lipid membranes uploaded with different target compounds are used as an innovative drug carrier for more effective and efficient ways to treat various diseases, such as cancers, inflammations, or immune-related diseases. This natural, environmentally friendly, safe, and affordable formulation, suitable for healthcare applications, can be developed with limited economic and environmental costs.

Perra et al. [94] studied the polyphenols extracted from grape pomace for their possible incorporation into phospholipid vesicles for intestinal delivery. The grape pomace was subjected to a slow extraction by maceration with ethanol, and phytochemical analysis identified malvidin-3-glucoside (55.8 mg/100 g), quercetin (67.0 mg/100 g), procyanidin B2 (98.1 mg/100 g), and gallic acid (43.2 mg/100 g) as major bioactive compounds. The grape pomace extract and soy lecithin were subsequently used as the basic components of the formulations. The researchers claim that phospholipid vesicles loaded with pomace grape extracts successfully protected Caco-2 cells (model of human colon-rectal intestinal epithelial cells) against oxidative stress, representing a promising delivery system for disease prevention and treatment [94]. Moreover, Manconi et al. [95] reported polymer-associated liposomes loaded with the grape pomace extract as a potential candidate for protection against oxidative stress in Caco-2 cells. Phenolics obtained from red grapes offered protection from degradation in the gastrointestinal environment, thus allowing the extract to exert its beneficial effect against oxidative stress at a cellular level [95]. Manca et al. demonstrated that polyphenols from grape pomace obtained from hydroalcoholic ultrasound extract and incorporated in liposomes are highly cytocompatible and can promote the proliferation of keratinocytes and fibroblasts [96]. In the case of a food product based on proteins and polyphenolic compounds, e.g., dairy beverages containing fruit juices, the interactions between these molecules may damage the product by causing precipitation and giving the product an astringent taste. This problem can be solved by the encapsulation of phenolics in the chitosan-coated liposomes, weakening the interactions between components [97]. The extract from seeds recovered from grape pomace, containing bioactive compounds such as epicatechins, catechin, gallic acid, quercetin, and procyanidins, was incorporated into phospholipid vesicles for intestinal delivery. The addition of maltodextrin allowed for remarkable resistance to acidic pH and high ionic strength, retaining the physicochemical stability of the nanovesicle, and counteracting the hydrogen peroxide-induced oxidative damage in Caco-2 cells [98]. Those nanovesicles (transferosomes and hyalurosome) improved the efficacy of different bioactive compounds or phytocomplexes.

### 3.2. Other Target Compounds from Grape Waste for Different Applications

The pharmaceutical and cosmetic industries are the primary beneficiaries of value-added products recovered from grape waste. However, grape wastes can also be used for other applications. The food industry can utilize wastes in different forms, including liquid, concentrate, or powder, focusing on all kinds of extracts exhibiting high antioxidant capacity, antibacterial properties, and delayed lipid oxidation [99]. Target compounds recovered from grape pomace can be used as food protectors due to their antioxidant ability to prevent lipid oxidation in fish-based products [100] and as replacers for synthetic antioxidants in pork meat [101] or lamb products [102]. Fibers obtained from pomace can reduce the rate of hydration and gluten development. Šporin et al. reported that incorporating grape pomace into flour (6%, 10%, and 15%, *w*/*w* flour basis) could increase wheat dough development and improve its rheological properties due to the changes in the dietary fiber content [103]. The soluble fibers may act as stabilizers in functional foods, preventing water mobility by forming a three-dimensional network with water, especially in dairy products [104]. Dietary fiber from grape waste has the property of gelling, thickening, and improving air incorporation in creamy products, such as ice creams [36]. Winery by-products have extensive uses, from possible prebiotic functions [105] to increasing the shelf life of seafood [106].

## 4. Challenges and Perspectives for Grape by-Products

Grape by-products are rich in phenolic compounds, but their recovery may sometimes be challenging. Grape secondary metabolites are perishable in the presence of oxygen, high temperature, elevated pH, and solvents, so the extraction parameters are decisive factors for their proper recovery [107]. Depending on their final application, the solvent used for extraction must be GRAS (generally recognized as safe), without showing any toxic effects. The use of non-toxic solvents with high-affinity levels and the optimization of extraction conditions can eliminate the toxicity problem, reduce extraction time, and lower the costs required to obtain the active ingredients [108]. The main challenge in the recovery strategy is developing methods with the capacity to simultaneously extract several ingredients. Due to the instability of the active ingredients, nano- and microencapsulation techniques are promising alternatives in this context. Biomass-derived nanomaterials are considered to have great potential for a diversity of applications due to new technologies being intensely developed.

Besides the beneficial health effects of grape waste-enriched nanomaterials, the toxicological issues should also be taken into consideration, the most important of which is the release of antimicrobials from enriched wound dressing materials or food packaging into the environment. There is also a high risk for the transfer of active ingredients from food packaging into food, leading to oral exposure and possible changes in natural intestinal microflora. Similarly, using silver or gold in nanomaterials can result in the release of metal ions and their transfer into the central nervous system [109]. Therefore, extensive studies are required to confirm the safety of all new applications before they are introduced for wide use.

The full implementation of the herein-described methods and approaches requires improving the knowledge of individual processes, from the characterization of wastes and their extraction to the final application. Implementing these processes on a pilot scale could also be the subject for further studies. The proper management of residues from the agro-food sector can turn valuable wastes into raw materials for other industries, thus increasing the economic competitiveness and resilience of the agro-industrial sector.

## 5. Conclusions

Plant-derived waste materials are expected to become effective therapeutic modalities for disease treatment, drug delivery, and other industrial or environmental applications. Agricultural or industrial waste-enriched nanomaterials can be obtained as metallic nanoparticles, polymeric nanocomposites, or lipidic nanopolymers. Metallic nanoparticles can be synthesized by bottom-up and top-down approaches. Fruit wastes represent a significant source of municipal waste and have been evaluated as reducing and capping agents in the synthesis of metallic nanoparticles. Due to their composition rich in phenolic compounds, these extracts possess the property of high nucleophilicity and can interact with metallic ions through chelation. The obtained metallic nanomaterials present antidiabetic, antioxidant, and antimicrobial properties, as well as the inhibition of carbohydrate hydrolyzing enzymes. Polymeric nanocomposites are of particular value due to their unique composition, based on nano-sized reinforcing agents, which have proven to be effective in enhancing biopolymer barriers and mechanical properties. These composite materials have desirable properties, such as lower toxicity risks, biocompatibility, biodegradability, recyclability, and tunable surface features, making them ideal for healthcare applications. The last class of discussed nanomaterials is lipid membranes, which, when uploaded with different compounds, can be used as innovative drug carriers targeting cancer, inflammation, or immune-related diseases. The development of these natural, environmentally friendly, safe, and affordable formulations, suitable for healthcare applications, can be achieved with limited economic and environmental costs. The primary applications of grape waste-based nanomaterials are pharmaceutics and cosmetics; nevertheless, the food industry, as well as the medical component and device industries, are also potential beneficiaries.

## Figures and Tables

**Table 1:** Short presentation of advantages and disadvantages of the pretreatment methods.

Pretreatment	Advantages	Disadvantages	Matrix for Which the Pretreatment Is Applicable	Reference
Hydrothermal	Does not involve chemical reagents	Decreased yield of lignin recovery	Materials with a poor lignin content	[20]
Irradiation	Depolymerization of cellulose and solubilization of lignin.	High costs	Woody materials with a high content of lignin	[21]
Alkaline pre-treatment	Solubilization of different compounds with high yields	Large amounts of water required for removing chemical compounds Large amounts of reagents required	Wastes containing lignin	[22]
Supercritical CO_2_	Low temperatures suitable for degradable compounds	Expensive process; special conditions required	Material with high lignin content	[23]
Biological method	Environment-friendly, low consumption of energy	Process is mild, with a slow rate of hydrolysis	Material with high lignin, cellulose, and polysaccharides content	[24]

**Table 2:** Different applications of materials obtained using *green methods*.

Nanomaterial	Plant Waste/Type of Extract	Extraction Method	Nanomaterial Properties	Application	Reference
Food packaging application					
AgNP	GSE/fresh juice	-	spherical; 23.8 nm and 12.1 nm zeta potential—−37.9 mV crystallite size—9.9 nm	Antimicrobial effect against A. niger ATCC16404 and *P. chrysogenum* T16 Materials were used as coatings to maintain the postharvest quality of grapes	[80]
	GPE/classical temperature extraction	100 mL of deionized water and heated at 60 °C for 10 min	spherical; 3 to 14 nm	Antimicrobial effect against *Escherichia coli* O157:H7 and *Listeria monocytogenes*	[81]
Environmental protection					
AgNP	GSE/fresh juice	-	average size of the AgNPs—54.8 nm	Reductive degradation of Direct Orange 26 in the presence of NaBH_4_	[86]
	*Vitis labrusca* pomace	150 mL of a hydroalcoholic solution (50% *v*/*v*); the mixture was centrifuged for 5 min at 5000 rpm and filtered	spherical and polyhedral shapes; 2.9–55 nm	Inhibition—75.3% for *Staphylococcus aureus* and 15.2% for *Enterococcus faecalis* in wastewater	[87]
Medical application					
ZnO NPs	Grape peels/aqueous extract	water extract (1:10), 50 °C, 1 h	nanocones, average size—19.36 nm	Bactericidal activity against *S. aureus* and *P. aeruginosa*; MIC—40 μg/mL; zone of inhibition (mm)—2–6	[88]
AuNP	Grape pomace/aqueous extract	2 g of grape waste in 50 mL of deionized water, boiled for 5 min	30 nm wide, having a cubic Au phase	antioxidants and tyrosinase inhibitors; sun protection cream	[83]
AgNP	Grape pomace/ethanolic extract	solid phase: ethanol—1:20	20–35 nm	Antioxidant and antibacterial properties (*Escherichia coli* and *Staphylococcus aureus*)	[84]
AgNP	Grape pomace/aqueous extract	aqueous-based solution at 80 °C for 4 h	face centered cubic (FCC) crystal structure; 15 to 20 nm	Antidiabetic, antioxidant potential, and antimicrobial activity	[85]
Nanocellulose/grape seed extract/AgNPs	Grape seed extracts -commercial	-	charred residue of TNC/GSE/AgNPs film—33.49% optical transmittance of 80–93% oxygen permeability (OP)—1.027 cm^3^ m^−2^·24 h^−1^·0.1 MPa^−1^	*E. coli*—zone of inhibition (mm)—2 *S. aureus*—zone of inhibition (mm)—6	[89]
Chitosan/alginate nanoparticles	Grape pomace extract	enzymatic extraction followed by freeze-drying (xylanase produced by *Aspergillus niger* 3T5B8 and Viscozyme^®^ enzymatic commercial cocktail—(from Novozymes Bagsvaerd, Denmark)	zeta potential (mV): −15 to −25.2 nanoparticle size—400 and 1000 nm	2-log reduction of *L. monocytogenes*, *P. aeruginosa* and *S. enteritidis*, and a 1-log reduction of *E. coli*	[90]

AgNP—silver nanoparticles; GSE—grape seed extracts; GPL—grape peel extract; MIC—minimum inhibitory concentration.
